# The inflammation, vascular repair and injury responses to exercise in fit males with and without Type 1 diabetes: an observational study

**DOI:** 10.1186/s12933-015-0235-y

**Published:** 2015-06-05

**Authors:** Daniel J West, Matthew D Campbell, Javier T Gonzalez, Mark Walker, Emma J Stevenson, Fahad W Ahmed, Stephanie Wijaya, James A Shaw, Jolanta U Weaver

**Affiliations:** Faculty of Health and Life Sciences, Northumbria University, Newcastle-upon-Tyne, UK; Institute of Cellular Medicine, Newcastle University, Newcastle-upon-Tyne, UK

**Keywords:** TNF-α, Endothelial progenitor cells, Circulating endothelial cells

## Abstract

**Background:**

Type 1 diabetes is associated with raised inflammation, impaired endothelial progenitor cell mobilisation and increased markers of vascular injury. Both acute and chronic exercise is known to influence these markers in non-diabetic controls, but limited data exists in Type 1 diabetes. We assessed inflammation, vascular repair and injury at rest and after exercise in physically-fit males with and without Type 1 diabetes.

**Methods:**

Ten well-controlled type 1 diabetes (27 ± 2 years; BMI 24 ± 0.7 kg.m^2^; HbA_1c_ 53.3 ± 2.4 mmol/mol) and nine non-diabetic control males (27 ± 1 years; BMI 23 ± 0.8 kg.m^2^) matched for age, BMI and fitness completed 45-min of running. Venous blood samples were collected 60-min before and 60-min after exercise, and again on the following morning. Blood samples were processed for TNF-α using ELISA, and circulating endothelial progenitor cells (cEPCs; CD45^dim^CD34^+^VEGFR2^+^) and endothelial cells (cECs; CD45^dim^CD133^-^CD34^+^CD144^+^) counts using flow-cytometry.

**Results:**

TNF-α concentrations were 4-fold higher at all-time points in Type 1 diabetes, when compared with control (*P* < 0.001). Resting cEPCs were similar between groups; after exercise there was a significant increase in controls (*P* = 0.016), but not in Type 1 diabetes (*P* = 0.202). CEPCs peaked the morning after exercise, with a greater change in controls vs. Type 1 diabetes (+139 % vs. 27 %; *P* = 0.01). CECs did not change with exercise and were similar between groups at all points (*P* > 0.05). Within the Type 1 diabetes group, the delta change in cEPCS from rest to the following morning was related to HbA_1c_ (*r* = -0.65, *P* = 0.021) and TNF-α (*r* = -0.766, *P* = 0.005).

**Conclusions:**

Resting cEPCs and cECs in Type 1 diabetes patients with excellent HbA_1c_ and high physical-fitness are comparable to healthy controls, despite eliciting 4-fold greater TNF-α. Furthermore, Type 1 diabetes patients appear to have a blunted post-exercise cEPCs response (vascular repair), whilst a biomarker of vascular injury (cECs) remained comparable to healthy controls.

## Background

Exercise carries many health benefits for people with Type 1 diabetes [1]. Of particular importance, regular physical exercise is associated with improvements in an array of cardiovascular risk factors such as cardiorespiratory fitness, endothelial function, and blood lipid profiles. These benefits are of significance given the raised cardiovascular risk and early mortality in these patients [2].

Exposure to hyperglycaemia and chronic inflammation ultimately means that individuals with Type 1 diabetes demonstrate raised markers of vascular damage, such as circulating endothelial cells (cECs; [3]), and endothelial dysfunction [4], in comparison with healthy controls [4, 3]. Accompanying this may be a reduction in circulating endothelial progenitor cell count (cEPCs; [4, 5]); bone marrow derived cEPCs are highly important for vascular repair and protection [5, 6] and are a significant predictor of endothelial function [4, 7] and cardiovascular risk [7].

A single exercise bout has been shown to acutely raise cEPCs count in both healthy and patient populations [8–11]. For example, Rehman et al [8] demonstrated that a single bout of cycling exercise significantly raised cEPCs by ~4 fold after exercise, in sedentary over weight males. Similar increases in cEPCs after exercise have been demonstrated in healthy, physically fit individuals, as well as in sedentary individuals with chronic disease [9]. However, data on the acute cEPCs response to exercise within Type 1 diabetes is lacking. Regular exercise training is associated with raised resting cEPCs and has a strong anti-inflammatory effect [12, 13]. Indeed, these benefits are likely integral to the cardio-protective role regular exercise provides. With this in mind, and the association of Type 1 diabetes with reduced cEPCs and raised inflammation, it is of scientific importance to determine the acute cEPCs response to exercise within Type 1 diabetes patients.

Moreover, how the resting cEPCs, cECs and inflammatory status of physically fit type 1 diabetes patients compares to matched, non-diabetic controls would also be of interest. Physical fitness is a measure that is not considered within the existing literature comparing cEPCs (marker of vascular repair), cECs (marker of vascular damage) and inflammation in this population [4, 3]. Therefore, the aim of this study was to assess the cEPCs, cECs and inflammation at rest and in response to submaximal exercise in physically-fit males with and without Type 1 diabetes.

## Methods

### Participants

Eligibility criteria for type 1 diabetes patients consisted of being aged between 18 and 35 years, a duration of diabetes > 2 years, and an HbA_1c_ < 8.0 % (64 mmol/mol). In addition, patients were required to be free of all diabetes-related complications including impaired awareness of hypoglycaemia (assessed via the Clarke method [14]) and not receiving any medication other than insulin. Participants in both the Type 1 diabetes and control group had to be regularly and consistently undertaking exercise (participating in aerobic based exercise for a minimum of 30 min at a time, at least three times per week, for > 12 months). Smokers were excluded from both groups. Ten males with type 1 diabetes and nine non-diabetic male controls, matched for age, anthropometry, and fitness were recruited for this study (Table 1). Patients were treated with a basal-bolus regimen composed of long-acting insulins glargine (*n* = 8) or detemir (*n* = 2), and rapid-acting insulin aspart. All patients were stable on their respective insulin regimen for a minimum of 1 year, and were familiar with carbohydrate counting, administering 1.0 ± 0.2 units of insulin aspart (IU) per 10 g of carbohydrate.

**Table 1:** Type 1 diabetes and control group participant characteristics mean ± SEM

	T1DM	CON	*P* value
Age (years)	27 ± 2	27 ± 1	=0.798
BMI (kg.m^2^)	24 ± 0.7	23 ± 0.8	=0.243
VO_2_max (ml.kg.min^-1^)	51.0 ± 2.1	50.7 ± 1.1	=0.808
HbA_1c_ (mmol/mol / %)	53.3 ± 2.4 /6.9 ± 0.2 %	---	---
Diabetes duration (years)	12 ± 2.0	---	---

*P* calculated from independent samples *t*-test

*BMI* Body Mass Index, *VO*_*2max*_ aerobic capacity

### Preliminary testing

Fully informed written consent was obtained from all participants following the study’s approval from local National Health Service Research Ethics Committee (13/NE/0016). Type 1 diabetes participants attended the Newcastle National Institute for Health Research Clinical Research Facility exercise laboratory for a preliminary screening visit as described previously in detail [15], before returning to establish peak cardio-respiratory parameters during the completion of an incremental-maximal treadmill running protocol as per the methods of Campbell et al [15], to determine the individual speed participants would run at during the experimental trial. The control group completed the same preliminary tests at the Exercise Physiology laboratory at Northumbria University.

### Experimental design

Type 1 diabetes patients were fitted with a real-time continuous glucose monitor (Paradigm Veo, Medtronic diabetes, Northridge, CA, USA) >24 h prior to the laboratory visit with high and low alerts set to help maintain glycaemia within normal ranges prior to the experimental session. Participants did not exercise within 96 h of the experimental visit.

On the experimental day participants were provided with standardised cereal-based breakfast and pasta-based lunch; prescribed by the research team. Participants arrived to the laboratory at 17:00 h. Following a resting blood sample participants consumed a pre-exercise carbohydrate bolus (corn flakes, peaches, semi-skimmed milk; 1738 ± 71 kJ / 415 ± 17 kcal) equating to 1.0 g.carbohydrate.kg^-1^ body mass. With this meal, patients self-administered a 25 % (2.0 ± 0.5 IU) dose (i.e. a 75 % reduction [15–19]) of rapid-acting insulin into the abdomen. Following this bolus, participants remained rested for 60 min, before commencing 45 min of treadmill running at a speed calculated to elicit 70 % of VO_2max_. This exercise intensity falls within current recommendations of the American College of Sports Medicine [20]. All participants completed the exercise protocol and there were no hypoglycemic episodes within the Type 1 diabetes group.

After exercise, participants remained at rest for 60 min before a further blood sample was collected. Participants were then discharged from the laboratory. On the following morning, participants returned to the laboratory at 08:00 h for a further resting, fasted blood sample.

### Blood sampling and data analysis

Venepuncture technique was used to collect 10 ml of whole blood at each respective sample point. After discarding the first four ml of collected blood, samples were evenly dispersed into a K2EDTA and lithium heparin tube. The lithium heparin tube was centrifuged for 15 min at 3000 rpm (4 °C) and stored at -80 °C for retrospective analysis of plasma Human TNF-α (Quantikine ELISA, R&D Systems, Roche Diagnostics, West Sussex, UK). The K2EDTA was sent for analysis of cEPCs and cECs immediately and was processed within 2 h [21].

### Circulating endothelial progenitor cells and circulating endothelial cells

#### Flow cytometry

100 μl of whole blood was incubated with 5 μl of V500 CD45 (BD Biosciences, Oxford, UK), 20 μl of PerCP-Cy5.5 CD34 (BD Biosciences, Oxford, UK), 5 μl of PE VEGFR-2+ (R&D Systems, Roche Diagnostics, West Sussex, UK), 5 μl APC CD133 (BD Biosciences, Oxford, UK), 10 μl FITC CD144 (BD Biosciences, Oxford, UK) for 30 min. Subsequently, 2 ml of pharmlyse (BD Biosciences, Oxford, UK) was used to lyse the red cells. The sample was then analysed by flow cytometry on BD FACS Canto™ II system and samples were run to approximately 500,000 total events. Analysis was carried out using BD FACSDiva™ software. On average 440,000 events were counted. CEPCs events were defined using the most recent definition of CD45^dim^CD34^+^ VEGFR2 (KDR)^+^, as recommended by Van Craenenbroeck et al. [21]. Intra-assay variation (CD45^dim^CD34^+^VEGFR-2^+^) was less than 8 %. The results were expressed as % leukocytes [22, 21]. CECs events were defined as CD45^dim^CD133^-^CD34^+^CD144^+^ [23, 24].

#### Statistical analysis

Statistical analysis was performed using PASW Statistics 18 software (IBM, Armonk, NY) with significance set at *p* ≤ 0.05. Plasma TNF-α responses were analysed using mixed model ANOVA (group*time) and are presented as mean ± SEM. CEPCs and CECs responses were assessed using two-way Friedman’s analysis and are presented as median ± IQR. Between group differences, and comparisons of the delta-change in cEPCs and cECs counts, were assessed using Mann-Whitney *U* test. Relationships were assessed with Pearsons product moment correlation coefficient or Spearman’s rank order correlation coefficient.

## Results

### TNF-α

**Fig. 1:**
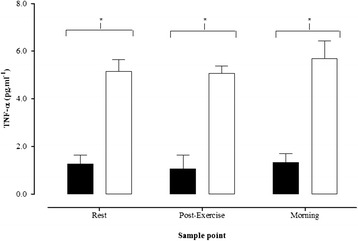
Plasma TNF-α at rest, 60 min post-exercise, and on the morning after exercise. Solid black bars = Control group, solid white bars = Type 1 diabetes group. * indicates a significant between group difference (*P* < 0.05). Data presented as mean ± SEM

Plasma TNF-α responses are presented in Fig. 1. There was no group*time interaction (*P* = 0.324), or change over time (*P* = 0.103), however there was a significant effect of group (*P* < 0.001). The Type 1 diabetes group elicited ~4 fold greater TNF-α concentrations at all sample points, when compared to control (Fig. 1).

### CEPCs & cECs

**Fig. 2:**
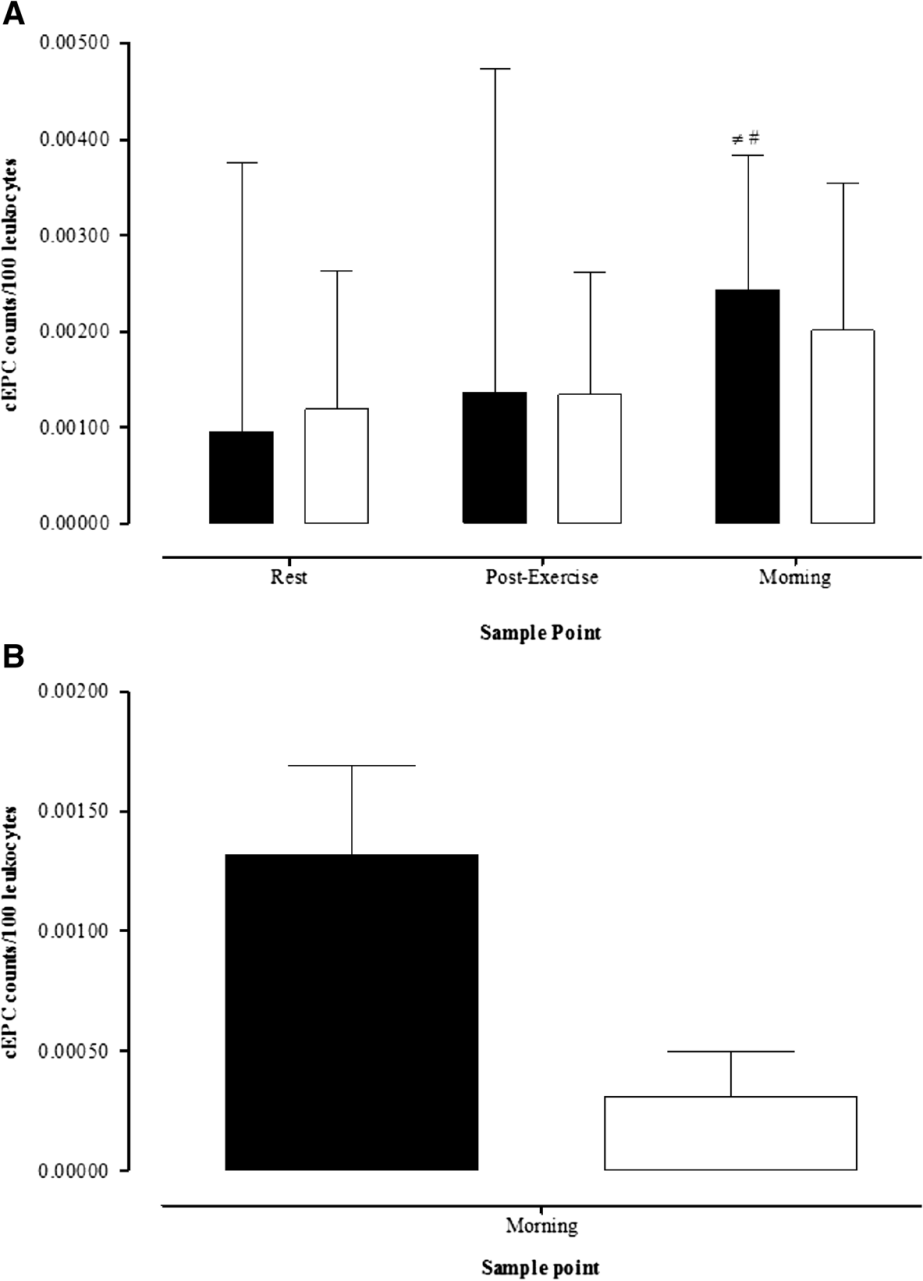
**a** Circulating endothelial progenitor cell (cEPC) counts at rest, 60 min post-exercise, and on the morning after exercise. Solid black bars = Control group, solid white bars = Type 1 diabetes group. ≠ indicates within conditions difference to pre-exercise, # indicates within condition difference to post-exercise (*P* < 0.05). **b** Change in cEPCs counts from rest to the morning after exercise. Solid black bars = Control group, solid white bars = Type 1 diabetes group. Data presented as median ± IQR

**Fig. 3:**
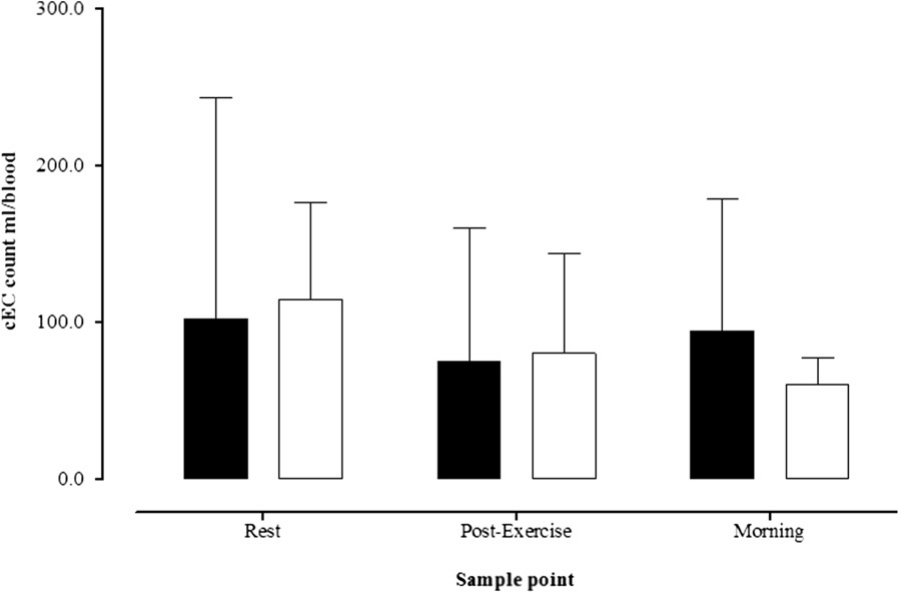
Circulating endothelial cell (cECs) counts at rest, 60 min post-exercise, and on the morning after exercise. Data presented as median ± IQR

CEPCs counts are presented in Fig. 2a. Baseline cEPCs counts were similar between groups (Fig. 2a). There was a significant increase in cEPCs counts after exercise within the control group (*P* = 0.016), however, there was no change within the Type 1 diabetes patients (*P* = 0.202). CEPCs counts peaked the morning after exercise within the control group (Fig. 2a) and the delta change, from baseline to morning, tended to be greater within the control group (Fig. 2b; *P* = 0.09). The median cEPCs count increased by 139 % and 27 % in the control and type 1 diabetes groups, respectively (*P* = 0.01). CECs count responses are presented in Fig. 3. CECs count was similar at all-time points between groups, and there was no significant change in cECs with exercise within either Type 1 diabetes (*P* = 0.179) or control (*P* = 0.236) groups.

### Relationships between variables

Within the Type 1 diabetes group, the delta change in cEPCS from rest to the following morning was related to HbA_1c_ (*r* = -0.65, *P* = 0.021) and TNF-α (*r* = -0.766, *P* = 0.005). There were no other significant relationships between cEPCs, cECs and TNF-α and participant characteristics (VO_2peak_, BMI, age and duration of diabetes) in either the control or Type 1 diabetes group.

## Discussion

This study aimed to examine cEPCs, cECs and TNF-α at rest, and in response to acute submaximal exercise, in physically fit males with and without Type 1 diabetes. This study is the first of this kind and we have shown that resting cEPCs and cECs were similar between groups, despite patients demonstrating ~4 fold greater TNF-α concentrations. However, the increase in cEPCs was blunted in the patients, with the change from rest to the morning following exercise being ~112 % lower in Type 1 diabetes, when compared to control participants. Furthermore, our data showed that this blunted response within the Type 1 diabetes group appeared to be predicted by HbA_1c_ and TNF-α concentrations.

### Resting cEPCs and cECs

Our finding that resting cEPCs and cECs were similar between groups is in agreement with Fadini et al. [25], but also contrasts with other literature in the area [4, 3]. In comparison with Sibal et al. [4], the Type 1 diabetes patients of this study were of comparable age and had similar duration of diabetes and also shared a raised inflammatory state. The difference in the findings are potentially explained by the high cardio-respiratory fitness and excellent glycemic control of our patients (HbA1c ~7 %, 53 mmol/mol vs. ~8.5 %, 69.4 mmol/mol [4]). Indeed, the participants in the study of Fadini et al. [25] also had good HbA_1c_ (~7.7 %). Whilst the relationship between glycemic control and cEPCs is established in Type 2 diabetes and acute myocardial infarction [26, 27], there are few studies in Type 1 diabetes. Most recently it has been shown that in children with Type 1 diabetes, cEPCs count was inversely related with HbA_1c_ [28]. In our study, it should be reassuring to both patients and clinicians that after achieving good glycemic control cEPCs were comparable to healthy controls. However, since our patients were also very fit, it would be interesting to investigate the change in HbA_1c_ and cardio-respiratory fitness both independently, and concomitantly (e.g. via an exercise training programme), to provide further insight into the normalised cEPCs we show.

### cEPCs response to exercise

This is the first study to examine acute exercise and cEPCs regulation in Type 1 diabetes and our findings suggest that regulation of cEPCs release in response to an exercise stimulus may be abnormal despite eliciting normal resting cEPCs and excellent glycemic control. Research examining the bone marrow biology of both animals and patients with diabetes confirms that bone marrow function is impaired [29] which may reduce cEPCs mobilisation [25]. Recent research has shown that the mobilisation of cEPCs in response to administration of recombinant granulocyte colony-stimulating factor is impaired in diabetes patients [25]. Speculatively, bone marrow function may also have contributed to the blunted cEPCs response to exercise demonstrated in the Type 1 diabetes patients.

It is well established that resting cEPCs are normal or high in active individuals [13, 9], thus it is of interest that both groups of participants in our current study had a cardio-respiratory fitness (VO_2max_ 50 ml.kg.min^-1^) which would categorise them as excellent, or above average (average VO_2max_ for males aged ~27 being ~ 42 ml.kg.min^-1^ [20]). Earlier research has described ~4 fold higher cEPCs in trained marathon runners when compared to sedentary control participants [13], and there is evidence that regular aerobic-exercise training is an effective intervention to raise cEPCs in both healthy [13] and patient populations [9, 30]. Potentially, the regular aerobic exercise engaged in by the participants in this study, evidenced by high cardio-respiratory fitness, may additionally explain the comparable resting cEPCs shown.

### Inflammation and cEPCs

Our study provides another interesting finding that the comparable cEPCs and cECs between groups were concomitant with ~4 fold higher TNF-α concentrations seen in our Type 1 diabetes patients. The raised inflammation is in agreement with our previous data of Sibal et al. [4]. There is evidence, in advanced cardiovascular disease patients, that cEPCs are reduced potentially due to the myelosuppressive effects of TNF-α [31]. Furthermore, three months of TNF-α inhibitory drug treatment has been shown to significantly raise cEPCs in rheumatoid arthritis patients [32]. Further research is required to investigate the interaction between the anti-inflammatory effects of regular exercise in populations which experience chronic inflammation, such as Type 1 diabetes.

We show for the first time that despite the excellent glycemic control and high physical fitness of the Type 1 diabetes patients there was a blunting of the rise in cEPCs elicited after exercise. Shear stress, increased nitric oxide (NO) production, through increased activity of endothelial nitric oxide synthase, and hypoxia are suggested to be key stimuli that contribute to the mobilisation of cEPCs with exercise [33, 9]. However, in some instances oxidative stress can reduce NO availability, which could, speculatively, attenuate the signal for cEPCs mobilisation. Recent research has shown that well controlled Type 1 diabetes patients demonstrate increased oxidative stress during aerobic exercise, in comparison with non-diabetic control participants [34]. Furthermore, there may also be a role for the myelosuppressive effect of TNF-α in these responses. Future research should examine the role of both oxidative stress and inflammatory signalling in the acute cEPCs response to exercise.

Although this study is limited by a relatively small sample size, it is important to consider the homogenous group that were observed, with patients all male, aged 19–34 years, HbA_1c_ range of 6–7.9 %, and all physically fit; as such, the importance of these data should not be underestimated. Future research should explore how improving HbA_1c_ independently of improving physical fitness influences markers of vascular repair at rest and in response to exercise. Conversely, it would be of interest to explore if improving physical fitness can rescue the deleterious effect of poor glycaemic control on cEPCs regulation. Furthermore, the role of the insulin species patients are treated with could be an additional factor to consider when examining the impact of exercise on cEPCs in this population [35].

## Conclusions

In conclusion, Type 1 diabetes patients with high cardio-respiratory fitness and excellent glycemic control present normal resting markers of vascular repair (cEPC’s) and injury (cECs), despite being in a persistent inflammatory state (raised TNF-α). However, the cEPCs response to acute exercise appears blunted, potentially limiting the cardiovascular benefits of exercise.

## Abbreviations

EPC: Response to exercise in Type 1 diabetes
cEPC: Circulating endothelial progenitor cell
cEC: Circulating endothelial cell
HbA1c: Glycated haemoglobin
TNF-α: Tumour Necrosis Factor Alpha
VO_2max_: Maximum oxygen uptake
